# ADP-dependent glucokinase: the ancient, archaeal key to prostate cancer

**DOI:** 10.1186/s40779-024-00514-x

**Published:** 2024-02-12

**Authors:** Marcin M. Kamiński

**Affiliations:** Department of Immunology, St. Jude Children’s Research Hospital, Memphis, TN 38105 USA

**Keywords:** ADP-dependent glucokinase (ADPGK), Prostate cancer, Prognostic marker, Adenocarcinoma, AMP-activated protein kinase (AMPK), Aldolase C (ALDOC)

The December 2023 issue of the *Military Medical Research* brings out an astounding discovery by Xu et al. [1] demonstrating a key role of the mysterious enzyme ADP-dependent glucokinase (ADPGK) in the cellular metabolism of prostate cancer (PCa). The ADPGKs are enzymes typically found in thermophilic archaea where they mediate the indispensable, first step of glucose metabolism, i.e. phosphorylation of glucose to glucose-6-phosphate. Strikingly, ADPGKs utilize ADP as a phosphate donor instead of ATP typically used to initiate glycolysis by four “classical” eukaryotic hexokinases (HK I–III and glucokinase). Thus, the discovery made by Ronimus and Morgan [2] of the functional form of ADPGK in mice, opened an intriguing question of the specific role of this enzyme in the metabolism of eukaryotic cell.

Now, the research of Xu et al. [1] showed that the unique mode of ADPGK metabolic activity is advantageous for the survival and homeostasis of PCa cells. Most importantly, ADPGK expression level could be used as a prognostic marker for the overall survival of prostate adenocarcinoma patients. Looking for a putative metabolic target for cancer treatment, the authors first analyzed the expression of all glucokinases in clinical tissue samples and correlated this data with predictions of clinical outcomes. Strikingly, ADPGK was not only overexpressed on protein and mRNA levels but also was the only glucokinase that predicted worse overall survival of PCa patients. Univariate analysis and multivariate Cox models showed that high ADPGK level was a risk factor predicting biochemical recurrence (BCR) for PCa patients. Next, the authors established the molecular mechanism underlying the role of ADPGK in carcinogenesis. Namely, modulation of ADPGK levels in PCa cell lines by overexpression or knock-down resulted in enhanced or decreased proliferation and migration, respectively. Moreover, using a subcutaneous xenograft model into BALB/c nude mice, the authors demonstrated that PCa cell line overexpressing ADPGK yielded tumors that grew larger and faster as well as had higher metastatic potential than control cells. Furthermore, through the utilization of metabolomics, proteomics and Seahorse respirometry, it was found that modulation of ADPGK level regulates the glycolytic phenotype of PCa cells, confirming the role of high ADPGK level for up-regulation of proliferation rate in PCa cells.

However, the most surprising findings were: 1) the identification that ADPGK regulates AMP-activated protein kinase (AMPK) activity, and 2) the molecular interaction of ADPGK with glycolytic enzyme aldolase C (ALDOC). Down- or up-regulation of ADPGK levels was sufficient to decrease or increase AMPK activity, respectively, while immunoprecipitation experiments showed that in PCa cells ADPGK binds ALDOC. In addition, ADPGK expression positively correlated with expression of ALDOC in PCa cell lines as well as in patient samples. Importantly, patients with concomitantly high levels of both enzymes had shorter BCR time than patients where one of the enzymes was low expressed. To verify their in vitro findings, the authors applied 8-Bromo-AMP, a pleiotropic ADPGK inhibitor [3]. The results of the treatment of PCa cells with 8-Bromo-AMP paralleled those of ADPGK knock-down. Thus, 8-Bromo-AMP emerges as a putative agent against PCa and further work on its analogues could result in more specific and efficient ADPGK inhibitors as means of treatment.

Since AMPK positively regulates glycolysis by phosphorylating phosphofructokinase 2, enhanced AMPK activation upon elevated ADPGK content could additionally contribute to an increase in glycolytic flux. It is, however, surprising that a knock-down of ALDOC expression in PCa cells decreased AMPK activity, as resulting lower glycolytic flux would be expected to promote AMPK activation as a feed-back mechanism for energy production. The possible explanation could stem from a higher relative functional importance of ADPGK than ALDOC for AMPK activation in a PCa setting. In the light of the recent findings by Xu et al. [1] several intriguing questions emerge. Firstly, in T cells, ADPGK was found to be transiently activated upon T cell receptor (TCR) triggering [4]. Interestingly, TCR stimulation induces also transient AMPK activation which timely coincides with this of ADPGK [5]. Since ADPGK generates AMP from ADP, ADPGK could potentially drive AMPK activation by elevating AMP/ATP ratio. Whether ADPGK-dependent elevation in AMP/ATP ratio contributes to ADPGK-mediated activation of AMPK in PCa cells or T cells could be a subject of further research [1]. TCR-mediated ADPGK activation contributed to the mitochondrial generation of reactive oxygen species (ROS) and subsequent nuclear factor-kappa B (NF-κB)-dependent transcription [4]. It is also intriguing to ask whether elevated ADPGK in PCa contributes to enhanced ROS production, and thus to pro-survival NF-κB-dependent transcription. In addition, finding that in PCa ADPGK is bound to ALDOC is reminiscent of a long-standing concept of metabolite channeling and “metabolons”, especially since in archaea ADPGKs are often endowed with additional phosphofructokinase activity.

Thus, although new open questions arise, the study by Xu et al. [1] constituted a very important step toward our understanding of how ADPGK regulates cellular metabolism in eukaryotes. Importantly, ADPGK was found to be a prognostic marker and a promising therapeutic target for PCa, one of the most aggressive and deadly tumors nowadays.

## Data Availability

Not applicable.

## Abbreviations

ADPGK: ADP-dependent glucokinase
ALDOC: Aldolase C
AMPK: AMP-activated protein kinase
BCR: Biochemical recurrence
HK: Hexokinase
NF-κB: Nuclear factor-kappa B
PCa: Prostate cancer
ROS: Reactive oxygen species
TCR: T cell receptor
